# Are codesigned programmes more difficult to implement? A qualitative study of staff perceptions on the implementation of a new youth mental health programme

**DOI:** 10.1111/hex.13989

**Published:** 2024-02-17

**Authors:** Michelle Kehoe, Rick Whitehead, Kathleen de Boer, Denny Meyer, Liza Hopkins, Maja Nedeljkovic

**Affiliations:** ^1^ Monash University and Alfred Health Melbourne Victoria Australia; ^2^ Alfred Health Melbourne Victoria Australia; ^3^ Centre for Mental Health and Brain Science Swinburne University Melbourne Victoria Australia

**Keywords:** codesign, early intervention, implementation, process change, youth suicide

## Abstract

**Background:**

Codesigned interventions are becoming more common in health services and, in particular, in the design and development of mental health programmes and interventions. However, previous research has established that the transition from codesign to implementation can experience several challenges and that this transition process has received little research attention.

**Objective:**

The aim of this study was to explore the experience of staff members charged with the implementation of a codesigned intervention for young people and adolescents at risk of suicide.

**Setting and Participants:**

Five staff members involved in the implementation of the new codesigned programme took part in semi‐structured interviews.

**Method:**

The study involved qualitative evaluation of staff experiences during the implementation of a new child and youth suicide intervention. Interviews were analysed using reflexive thematic analysis.

**Results:**

The analysis identified four themes of ‘disconnect’, ‘operational challenges, ‘service user’ and ‘being authentic’. ‘Disconnect’ captures the difficulties of implementing a codesigned programme which leads to ‘operational challenges’ in meeting broader expectations while ensuring the feasibility of the programme. The third theme, ‘service user’, captures the realisation that the young people accessing the new service were different to those involved in the codesign process. The final theme, ‘being authentic’, highlights how staff needed to be responsive and flexible while remaining true to the principles proposed in the codesign.

**Discussion:**

This study yielded some valuable insights into the challenges around the implementation of a codesigned intervention, an under‐researched area. The findings suggest that adaption of the design may be necessary, if it is not informed by implementation constraints, making it necessary for the implementation team to be well‐briefed on the initial design and given plenty of time to make the necessary adjustments in a coproduction process. Limitations for the generalisation of the results include a small sample of staff and particular challenges that may be unique to this study.

**Conclusion:**

The present study highlights that for health services undertaking codesign approaches, appropriate time and resources need to be considered for the implementation phase of an initiative, to ensure that there is effective translation from design to implementation and that new codesigned services can be effective within operational constraints.

**Patient and Public Contribution:**

The authors would like to thank and acknowledge the young people with a lived‐experience and their carers who participated in the codesign process and research evaluation component of this study. We also wish to thank the clinical staff, peer workers and family peer workers who participated in the evaluation.

## 1 INTRODUCTION

Suicide is one of the leading causes of death in children and adolescents, making this a priority area for public health interventions in many countries.^1^, ^2^ To tackle the growing incidence of youth suicide, there is an increasing recognition of the importance of including the input of service users in the codesign of suicide prevention and intervention strategies.^3^

The use of codesign approaches is a means to address power differentials and minimise epistemic inequality.^3^ Despite research showing the benefits of including the voice of service users in suicide prevention programmes,^3^ there can be challenges when translating the outputs of a codesign process into practice. This can be particularly challenging when there is a tension between the ideas formulated in the codesign process and the need to make a final decision around what can realistically be implemented.^4^ While the implementation of new programmes in public health is often challenging,^5^ the implementation process may be even more complex when the intervention has been designed with input from a variety of stakeholder perspectives. Issues identified through the process of codesign, involving both lived experience and professional perspectives, may be challenging to translate into practice within the context of existing service models, bureaucratic reporting and performance measurement requirements and unspoken assumptions about the importance and effectiveness of traditional or existing models of care.^5^, ^6^

A recent systematic review identified the need for communication, training, support and flexibility as particularly important factors for the successful implementation of codesigned interventions.^7^ However, research in this space has been limited, leaving a gap in the literature. This paper seeks to address this gap by presenting the findings of an evaluation into the experiences and views of a team of mental health professionals charged with implementing a new programme to address youth suicidality. The new programme was commissioned and funded by the local Department of Health, and was codesigned through an iterative process of workshops involving young people who had previously used youth mental health services, parents/carers of young service users, professional staff of the mental health service where the new programme was to be embedded, and staff members of key stakeholder organisations with connections to the mental health service and other local community supports. The findings from the codesign workshops are described in Section 1.3.

### 1.1 Codesign in mental health services for young people

Collaboration with individuals who use programmes and initiatives has become increasingly common in the design of mental health services over the last decade.^1^ The concept of ‘design *with* consumers’ rather than ‘design *for* consumers’ has been a critical component of this development.^2^ Involving a range of stakeholders, such as health service staff, government departments, community members *and* those with a lived experience of service use, has been found to create services that are more comprehensive, accessible and credible than services which do not include such views.^3^, ^4^

A full cocreation process takes a broad collaborative approach, involving multiple stakeholders for the identification of a problem, generation of a solution, implementation of the design and evaluation of the outcomes.^8^ In contrast, codesign encompasses the active process of collaboratively designing solutions to a prespecified problem, often in a group workshop setting.^8^, ^9^

Despite the positive outcomes that may be associated with the codesign of services, several barriers and limitations have also been identified in prior codesign processes.^6^, ^10^, ^11^ For instance, Pirinen et al.^6^ identified barriers to an effective codesign process, such as: conflicting goals and expectations; complexity of organisations; existing processes in real‐life contexts; systemic resistance and professional power hierarchies; and lack of ownership and leadership for the codesigned intervention.^6^

While acknowledging and accounting for the challenges associated with the codesign stage of interventions, understanding the processes of implementation of such interventions is equally important. Even if the codesign phase follows best practice, and yields important ideas and opinions from multiple stakeholders, the knowledge of and input from these multiple perspectives can be lost if not effectively translated during programme implementation.^6^, ^12^ The gap between designing effective services and delivering effective care highlights a paucity in knowledge and research around the implementation process which must follow a codesign process.^13^

### 1.2 Implementing codesigned interventions

According to implementation science literature there are many factors that can affect implementation, including stakeholder perceptions of acceptability, ability to maintain an effective initiative or programme at a sufficient level of quality, new practices being added on top of existing ones rather than being effectively change managed, and overload, leading to ‘slippage’ such as diminished staff conditions or lack of resources.^13^, ^14^, ^15^

However, there are additional barriers commonly associated with the implementation of coproduced interventions. For instance, Larkin et al.^5^ found that when project team members or champions leave or change roles, continuity of care and implementation success may be negatively affected. They also found that when projects move from the design to the implementation stage it can result in a small number of individuals being placed under increasing pressure but with limited powers to create change.^5^ Other issues regarding the implementation of coproduced programmes concern the governance associated with the implementation process, including factors such as a lack of adequate communication between the various stakeholders, a lack of management support, and staff feeling torn between clinical duties and the implementation process. These issues highlight the importance of ensuring that the right supports and resources for implementation are available and sustainable.^5^

Prior research also suggests that following a codesign process if not provided with any implementation guidelines, clinicians tend to make new implementation choices based on their prior experience of what has worked in the past.^16^, ^17^ This approach may have limited success for the implementation of a new codesigned intervention, since prior implementation approaches may be a poor fit for interventions designed with different ends in mind.^6^ Kirk et al.^18^ suggest a balance between existing contextual knowledge and research evidence for informing an implementation process. This avoids putting an implementation at risk because too much emphasis has been put on pragmatic justifications of what staff ‘believe’ would work well because it has worked in the past. Instead, it is argued that implementation strategies should be based on a purposeful, specific course of action, in line with the codesigned model rather than previous routines, habits or prior experiences.^17^

### 1.3 Background for this study

Reviews of the current mental health system in Australia, and the state of Victoria in particular, have highlighted the need to consider how mental health services are designed, implemented and delivered.^10^, ^19^, ^20^ The 2023 Victorian Royal Commission into the Mental Health System reported that the system required significant reform.^19^, ^20^ A major recommendation of the commission was to embed codesign into mental health interventions and treatment programmes. The intervention referenced in this paper is an intense, psychosocial out‐reach model of care, designed for young people aged 12–26 with persistent suicidal ideation or postsuicide attempt. The model of care was codesigned specifically for young people by a range of stakeholders, following the implementation of a similar adult model of care.^21^ Details of the codesign process evaluation are reported elsewhere.^22^ However, the outcomes from the codesign workshops are briefly described below.

### 1.4 Codesign findings and features of the new service

There were two codesign workshops conducted by the project team. Workshop 1 consisted of a brainstorming session by all stakeholders on the ‘ideal’ features of a postsuicide programme for young people. In the second workshop, the project team and stakeholders sought to translate the ‘ideal’ programme features into a map of the proposed service provision for a typical young person.

The main findings from the initial codesign workshop focused on the qualities of the staff, the involvement of family members, and service accessibility. Participants in the codesign discussed the need for a nurturing, human‐centred approach to care that involved peer workers with lived experience. Participants also identified the need for the service to provide support for parents and families through parent education and family therapy, although it was also deemed important that the young person was given control in making decisions about what was best for them. A further outcome of the codesign was the need for a service with easily accessible, in‐person care with a continuity of staff to avoid the young people having to repeat themselves.

Subsequently, the purpose of the second workshop was to use the agreed themes from the first workshop to create a model of care. The second workshop included the project team and the same Workshop 1 stakeholders. The model of care was mapped by simulating the journey of a fictional young person. ‘typical’ of those expected to use the service. The mapping process involved detailing *entry* into the service (following referral), the *episode of care* and *departure* from the service. The second workshop identified four key characteristics that the new service needed to feature:

1. Contact from CY (Child and Youth) Hope team with 24 h of referral.
2. Initial meeting with the young person's support network, assessment and safety plan within 3 days of referral.
3. Collaborative, psychosocial, intense, outreach care.
4. 3 Months period of engagement.

A new CY Hope team was established to implement this model of care, consisting of a mix of mental health clinical staff (psychiatry, psychology and allied health), psychosocial support staff and lived experience staff (young person and parent/carer peer workers). The team accepted referrals for young people aged under 25 years who had had a suicide attempt or experienced persistent suicidal ideation. Referrals came from a range of health care providers, including hospital inpatient units, crisis assessment and treatment team, private providers and self‐referral. The service provided included an intensive, time‐limited (3 months) outreach model of ‘wrap around care’ to the young person and their family (where possible) or other members of their immediate support network (extended family, intimate partner, etc.). Support is tailored to each individual and could include a mixture of medical (e.g., medication review), psychological, psychosocial (e.g., financial, housing, education and employment support) and peer support, as well as a warm handover to ongoing care following discharge from the programme.

### 1.5 The focus and aim of the current study

The aim of this study is to examine the experiences of a team of mental health care staff charged with implementing a new co‐designed model of care. In particular, this involved the need to incorporate both the higher level ‘ideals’ from codesign Workshop 1 and the practical details of the proposed model of care from codesign Workshop 2 (see Section 1.4). Participants were invited to reflect on the implementation process, with a particular focus on the ways in which implementing a codesigned model differed from their usual ways of doing things.

## 2 METHODS

### 2.1 Study design

The study used a qualitative design with semi‐structured interviews, following questions that were designed and agreed to by the research team. The study used this methodology since it sought to understand the experiences of participants in their own words.^23^ See Appendix A for the interview guide.

### 2.2 Participants

All staff (*n* = 5) involved in the implementation process took part in the study. Two of these staff members were involved in the codesign process. The remaining three were newly employed at the service and were expected to implement the codesigned model without having taken part in the codesign process. Staff interviewed consisted of two youth peer workers (staff members under the age of 26 with a lived experience of mental health challenges), a family peer worker (a staff member with an experience of caring for a young person with mental health challenges), and two clinical staff. See Table 1 for demographic data.

**Table 1:** Participant demographic data.

Role	Years of experience in role	Specialist mental health trained	Participated in codesign workshop
Youth mental health peer worker	Less than 5	Yes	Yes
Youth mental health peer worker	Less than 5	Yes	No
Family peer worker	5–10 years	Yes	Yes
Mental health allied health clinician	15–20 years	Yes	No
Mental health allied health clinician	Over 20 years	Yes	No

### 2.3 Procedure

Interviews occurred in June 2022, approximately 4 months after the programme implementation commenced. Participants were initially contacted via email to obtain an expression of interest to participate and to provide consent to be interviewed. On ethical advice, participants were advised that their comments would not be linked to themselves or their role within the team. This was done to ensure anonymity and to reassure participants that they could freely express their views. Interviews were conducted by telephone or videoconferencing and were audio recorded. Interviews ranged from 48 to 61 min in duration.

Thematic analysis of the transcribed data was conducted by various members of the research team using the six‐step thematic approach recommended by Braun and Clarke.^23^ Initially, this involved the research team immersing themselves in the transcript data. After this, transcripts were given a broad initial code with a description for each code. These codes were refined iteratively by two researchers (M. K. and R. W.) until they could be refined into themes and subthemes. Finally, quotes were extracted to represent the themes. Ethics approval was obtained from Alfred Health.

## 3 RESULTS

Results are presented with main themes, codes, and example quotes which were developed inductively as described above. A summary of the main themes with broad descriptions is shown in Table 2.

**Table 2:** Summary of themes with a broad description.

Theme	Code/description
Disconnect	Challenges for staff implementing a new ‘ideal’ programme within the scope of the hosting service.
Operationalization challenges	Seeking to meet the expectations of stakeholders, without a model to emulate, while ensuring feasibility.
Service user	The realisation of staff that those accessing the service was different to those involved in the codesign and differed from expectations.
Being authentic	The need for staff responsiveness and flexibility in making adaptations while ensuring they stayed true to the codesign.

The interview data revealed a range of interlinked themes which flowed out of staff members' experiences while undertaking the implementation process. The first theme related to a disconnect between the idealised codesign outputs and the practical requirements of implementation. This theme of disconnect describes the difficulties of implementing a new programme from an operational perspective, and the disjuncture between what had been created through the codesign process and the realities of what the hosting service could offer, given the existing constraints of workforce shortages, tight budgets and an unexpected new cohort of young people presenting for care.

For staff, this meant attempting to manage these competing demands while accommodating various needs (Table 3, Quote 1). All of the participants commented on the challenges with meeting funder requirements, such as key performance indicators (KPIs). For example, ‘it would make it tricky initially … really hard to …. meet those KPIs’. Another person commented on the volume of training required by the funder and felt that the excessive requirement for training was designed to address *any* perceived risk (Table 3, Quote 2). Yet, there was an acknowledgement that ‘at times, it felt we were having to accommodate their [the funder's] needs rather than the needs of the service’.

**Table 3:** Main themes and example quotes.

Theme	Data
Disconnect	*Quote 1*. We're working really hard to accommodate everybody's wishes. *Quote 2*. I think for me … one there were. lots of requirements from the biggest breakdowns is probably people not having[funder] as well … I think part of the time to understand what the co‐design was reason why I was so inundated with training is because of this worry of risk.
Operational challenges	*Quote 3*. Significant delay between we're [as a service] not at the co‐design finishing and commencement of the implementation point where team leaders understand lived‐experience. *Quote 4*. [there has] been this sort of disconnect … the clinical work is just traditional … (while) lived experience (although) being in the team, but the functionality of it by default is they're outside the team.
Service user	*Quote 5*. The majority of our referrals have been 18 to 25 years … who don't live with their family because their family might be part of the problem. *Quote 6*. When we first started, it was pretty much all international students …. and so those network meetings and all those things were just impossible to do because, they (the young consumers) all lived by themselves.
Being authentic	*Quote 7*. I think they're like some really key qualities to have … in terms of creating a service like this is having people that are really open‐minded and willing to be vulnerable and open to change and um, being adaptable and flexible. *Quote 8*. We've still been able to sort of collectively provide a really good service to clients … and appreciate that in each other, despite the disagreements around we should do it this way or that way.

This disconnect can epitomise the challenges many services face when managing the competing demands of delivering an intervention while also meeting the requirements of those they report to. It also highlights the disconnect in expectations of the various stakeholders. As a result of their experiences, the staff pondered on the ‘relevance’ of the programme that was to be created. Ultimately, the staff implementing the new programme felt that the ideals identified in the initial phase of the codesign were ‘concepts [that were] too big and bold' 'not realistic' and 'not achievable'.

In addition, a delay of several months between the completion of the codesign process and the employment of programme staff, as well as significant staff turnover within the programme, resulted in challenges in transforming the codesign outcomes into an operationalizable model. This disconnect led to the second theme found in the evaluation data, which was the operationalization challenges. Staff felt there was a level of expectation regarding what the service model would entail or look like, which did not fit with their own experience of what was feasible. This was also related to a lack of information on what this new service would or should look like, since there was no template or similar model they could emulate.

One staff member explained ‘a huge part of it is that there are no major documented processes’ and ‘there wasn't any general agreement’. The codesign outputs focused on broad principles, such as the timeliness of response, without addressing practical issues such as eligibility criteria or staffing requirements to meet the demands. For example, the staff felt there was no understanding of ‘the referral process’ or who staff were expected to ‘screen in’ or ‘screen out’ to another service.

Although the desired outcomes from the codesign workshops were articulated, staff struggled because the ideas were ‘not operationalized anywhere’ and they felt that any process they followed was ‘just random’. In addition, the use of young peer workers, with lived experience of mental health concerns, was a new concept for the team. Staff felt that as a service, the extent of knowledge about working with lived experience staff was limited and this was particularly evident at the leadership level (Table 3, Quotes 3 and 4). This issue was considered as adding another layer of complexity to the implementation process (Table 3, Quote 5). Despite this, there was an understanding from the codesign workshop that the young peer worker would be ‘at the front’ of the service model, meaning that they would act as the first point of contact for those entering the service. This meant that the only youth peer worker in the team would need to ‘make contact within 24 h’. However, this could not occur until ‘someone clinical’ had examined the referral for suitability to the service first. As a result, this created ‘frustrations around the timeliness of people making those calls’. These problems highlight the need for clear processes to be codesigned along with the model of care, so that the outcomes from codesign workshops have a greater chance of being successfully implemented.

However, despite these problems, staff were able to reflect on what the process had taught them; you ‘can't rush implementation’; the need for a ‘team working together’; ‘training’ before commencement; and greater ‘clarity’ around expectations. These views indicated that, despite the challenges faced by staff, there was an ability for them to separate the issues that were not within their control and could not be changed, from issues within their control which they could adapt to suit the new programme.

The third theme to be identified from the data was that of the ‘service user’ and the realisation that the cohort of young people presenting to the new service represented a different demographic than the young people and families who had been involved in the codesign process, and the ‘typical’ fictional person utilised in the second codesign workshop. Following the commencement of the new initiative, staff described several issues around their expectations of the young people referred to the programme. The anticipation and expectation of staff was that the referrals would be from a similar demographic to users of the service that they were familiar with. One staff member reflected ‘I don't know what they [the funder] had in mind’ since the referrals did not come from what staff considered as being a ‘typical’ service user, which was described as being ‘an intact family and a big house’. Instead, staff found that referrals tended to be young people who didn't live at home, perhaps due to the family being a part of the issue or because the young person was an international student with no family nearby (Table 3, Quotes 5 and 6). As such, staff felt there was a distinct referral mismatch in what they expected, had been led to believe, and also what was identified through the codesign process or consultation with the funder.

Furthermore, the codesigned model of care sought to include families and significant others at ‘network’ meetings with the child or young person. However, it soon became evident that this model would not be viable for a large percentage of the cohort seeking care (Table 3, Quote 6). A key aspect of the model of care recommended in the codesign process was the use and need for out‐reach services for young people. However, this unexpected cohort of young people also brought practical issues in conducting outreach, when there was more than one staff member in attendance; ‘if you tried to take out three [staff] people [to see the client], somebody would have to be in the hallway because they lived in student accommodation’.

The experience in implementing the model led to the fourth identified theme ‘being authentic’, which is the need for flexibility, adaptability and responsiveness in implementation, while staying true to the principles of what was proposed in the codesign. The temptation for staff may have been to revert to traditional models of working, however, it is clear that with supportive leadership and commitment to the process, these challenges were overcome. Further to this, the staff felt they had built a good team who were ‘open to change, adaptable and flexible’ when they needed to address the challenges faced (Table 3, Quote 7). Despite some ‘disagreements’ among staff around the way things should be done, overall they all believed that the young people received a really ‘good service’ that was ultimately running ‘smoothly’ and that provided excellent ‘wrap‐around support’ to the young consumers (Table 3, Quote 8). Staff were able to maintain fidelity to the overarching principles of the codesigned model, while tweaking the implementation to meet the needs of both the funding/reporting body and the young people at the centre of care.

## 4 DISCUSSION

This study examined the implementation of a new programme to support children and young people at risk of suicide. The aim was to understand the experiences of staff charged with implementing the programme in accordance with the recommendations from the codesign process.

While codesign is used primarily to develop interventions that are best suited to the users of the service,^23^ this also raises implementation challenges around relinquishing power by some professionals, managing service level constraints regarding funding, staffing, KPIs and resource management and risk mitigation when involving those with suicidal thinking.^24^

There are particularly challenges for implementation staff in developing a desired programme within existing operational requirements. The concept of ‘disconnect’ is indicative of everyday implementation issues, as a struggle is enacted between idealistic models and real‐life operational contexts.^6^ These issues can be exacerbated when nominated ‘champions’ leave or move roles, which can result in a lack of continuity.^5^ This may be even more significant when the model has been codesigned rather than created solely by professionals, as continuity of vision between the codesign participants and the service delivery staff can be easily lost. The model which results from the codesign process may not be adopted if implementation staff only consider methods that have worked in the past, believing that the old ways are more suitable,^5^, ^17^, ^18^ or being unfamiliar with the process by which the model has been developed.

Prior research has found that codesign processes are not only complex but can also often only partially translate into successful implementations.^24^, ^25^, ^26^ It has been reported that the implementation of codesigned interventions within existing frameworks is particularly difficult due to the complexities of creating relationships, understanding stakeholder roles and needs, and unrealistic stakeholder expectations.^23^

In addition to unmet staff expectations, there were also unrealistic expectations and conflicting demands from the funder and the health service responsible for the implementation, described by staff as ‘competing demands’.^25^ Similar findings were highlighted by Cosgrave et al.^26^ who found a number of challenges associated with balancing the needs of mental health programmes with the expectations of the funders. They suggested the need for very clear communication, and an ongoing two‐way process, whereby all stakeholders are actively engaged throughout all stages of the design and implementation process. This can allow stakeholders to discuss factors such as changes in understanding relating to targeted demographics, structural frameworks for programme delivery and the management of expectations of funding bodies. This should ensure that the codesign‐to‐implementation process remains an iterative process, where changes can be implemented based on any new information that emerges.^4^, ^26^ Clearly, implementation is not a linear process with a clear beginning and end. It is often a complex, lengthy change process. Hence, the above strategies are needed to ensure long‐term success.^15^

Prior research has also reported that it can be challenging for a small team of staff to undertake implementation of a new programme when they have limited powers to make changes to the model of care.^5^ This challenge is not unique to small teams and is commonly reported in codesign projects.^5^, ^27^ Team size can often diminish during the course of a project for reasons such as high staff turnover, lack of management support, feeling overburdened, and staff doubts over sustainability.^28^, ^29^ Each of these challenges can result in cost implications for a project which may not have been accounted for during the codesign phase.

To address the disconnect when moving from the codesign to implementation stage, there ideally needs to be consistency in staffing, and a true coproduction process which also takes account of evaluation. Coproduction should not be considered as a single linear event but one of a series of actions that brings together stakeholders at each stage of the journey from the original concept through to evaluation outcomes.^16^, ^30^ In addition, there is a need for clearer documentation around processes and procedures to provide consistency as well as to reduce gaps in knowledge. Monitoring the process in the early stages of implementation is critical to providing robust evaluation, not only in relation to the clinical outcomes of the intervention but also around fidelity during the implementation of the programme.

In the current study, it should also be noted that there was some minor difference in understanding between those who had participated in the codesign process and those who had not. However, this may relate to the different roles played by each team member and/or the small sample size. We cannot be confident that this finding is broadly applicable or indicative only of this particular study. Regardless of how well constructed the final codesigned model is, implementation staff who experienced the full codesign process are likely to have a deeper understanding of most aspects of the model than staff who are employed only at the point of implementation. Employment of some staff at the point of implementation may also inadvertently create tension within the team.

The present study highlights that for health services undertaking codesign approaches, appropriate time and resources need to be considered for the implementation phase of an initiative to ensure there is effective translation from design to implementation and that new services can be effective within operational constraints.

The current study builds upon the limited research examining the difficulties with implementing codesigned approaches, and contributes novel insights and considerations for future codesigned programmes. Indeed, while the process of codesign is often reported, Bray et al.^31^ found that the experience of implementation of codesigned interventions is seldom reported. In those that do report on the implementation process, factors such as lack of team communication and support,^32^, ^33^ and team member burden, such as juggling other responsibilities,^34^ were barriers to the implementation, while effective team structures^33^ facilitated the implementation of codesigned programmes. Other facilitators for the successful implementation of codesigned programme which have previously been identified by De Boer et al.,^7^ include promoting codesign outcomes at implementation and flexible delivery approaches. This study has shown that these facilitators were present in this implementation as well, resulting in a ‘really good service’.

A strength of this study is its complete coverage of the views of the implementation team, ensuring that a balanced view is obtained across all roles. In addition, there was an honesty and willingness to disclose that can perhaps be attributed to the trustful relationship developed between the researchers and the participants.

### 4.1 Limitations

Beyond the positive aspects of the study, there are a number of limitations present. First, the small sample size in the present study raises concerns regarding the power of the data and whether data saturation can be reached with a sample of only five.^35^ However, the current sample consisted of everyone involved in the implementation process and as such, it was not possible to have a larger sample size. Further, due to the specificity of the sample, the in‐depth nature of the interviews, and the thematic analysis process, the present sample was considered to have sufficient ‘information power’ to yield meaningful results.^36^

Additionally, only limited demographic data, unlinked to quotations, was presented for the sample. This decision was made to preserve anonymity of the sample and to ensure that all participants would feel that they could talk freely about the implementation process. Future studies with a larger sample size and more demographic information may provide more generalisable and contextual data.

The study was also conducted quite early in the implementation process. It would be interesting to conduct a follow‐up study at some later time to determine the extent to which fidelity to the codesigned model has been observed, or, conversely, regression to the traditional way of operating has occurred. A further limitation was that the study only included members of the implementation team, and was conducted by professional researchers. The inclusion of a lived experience perspective in the research team, and the inclusion of study participants with other perspectives such as service management may have provided different perspectives on the implementation process.

## 5 CONCLUSION

The current study adds new knowledge to the limited research around the implementation of codesigned programmes.^6^ In particular, it demonstrates the challenges faced by an implementation team that was required to balance fidelity to the codesigned model with the bureaucratic requirements and practical constraints of operating within a public mental health service. Themes such as the disconnect between the codesign process and the implementation setting, along with operationalization challenges due to lack of guidance around process, illustrate areas where more work needs to be done to ensure codesign can be effectively embedded in service delivery. Recognising that in some cases the service that has been designed may not be congruent with the cohort of clients presenting for care, and incorporating sufficient flexibility to adapt to these kinds of situations, are key requirements for successful implementation.

Future studies evaluating implementation processes for codesigned interventions should seek to gain regular feedback and opinions in an iterative manner to ensure that programme redesign takes account of operational realities. In addition, a triangulated approach to data collection which includes other stakeholders within the implementation, such as the funder, other staff in the mental health service, and the young people using the new service would be of value to tease out and understand additional nuances.

## AUTHOR CONTRIBUTIONS

**Michelle Kehoe**: Conceptualisation; investigation; funding acquisition; writing—original draft; methodology; validation; writing—review and editing; formal analysis; project administration; resources; data curation. **Rick Whitehead**: Writing—original draft; writing—review and editing; validation; formal analysis. **Kathleen de Boer**: Writing—original draft; writing—review and editing. **Denny Meyer**: Funding acquisition; writing—review and editing; formal analysis. **Liza Hopkins**: Writing—review and editing; project administration; formal analysis. **Maja Nedeljkovic**: Formal analysis; writing—review and editing.

## CONFLICT OF INTEREST STATEMENT

The authors declare no conflicts of interest.

## ETHICS STATEMENT

Ethical approval for the study was obtained from the Alfred Health and Swinburne University Human Research Ethics Committees. All participants were older than 17 years of age and provided written consent.

## Data Availability

The data that support the findings of this study are available from the corresponding author upon reasonable request.

## APPENDIX A STAFF INTERVIEW

You have been invited to participate in this interview so we can understand the opportunities and challenges during the implementation of the new service, the resources, how the service was received by others, and what need to be improved. We would like to record this interview with your permission. This recording will not be made available to any other members of the Alfred Child and Youth Hope team.

1. What is your current role in the Alfred child and youth Hope team?
2. How long have you been in the role?
3. How did you come to be involved in this team/what appealed to you?
4. What were the primary challenges you or the team faced during the implementation of the CY HOPE project?
5. What were the primary facilitators to implementation?
6. What resources and supports did you have access to during the implementation? Were these resources adequate? If not, what resources were missing?
7. What training did you receive?
8. What type of additional training would have been helpful?
9. Do you think the service needs to be expanded?
10. If so, what needs to be taken into account (e.g., longer hours, more staff etc)?
11. What do think the benefits of CY Hope are

a. For young people
b. For families
c. For yourself
d. For other staff members?

12. Do you think there could be any disadvantages from having CY Hope at Alfred CYMHS?

a. Prompt if needed: takes time/resources from other programmes

i. Not sufficiently medical/treatment based
ii. Could be traumatic for the CY Hope workers
iii. Could be not relevant for the young person
iv. Could distract from attendance at medical/mental health service

13. Is there anything else you would like to share with me?
